# Thygeson’s superficial punctate keratitis (TSPK): a paediatric case report and review of the literature

**DOI:** 10.1186/s12886-020-01790-6

**Published:** 2021-01-29

**Authors:** Xiao-Jiao Tang, Qing Liu, Lian-Hong Pi, Xin-Ke Chen, Lin Chen

**Affiliations:** Department of Ophthalmology Children’s Hospital of Chongqing Medical University, National Clinical Research Center for Child Health and Disorders, Ministry of Education Key Laboratory of Child Development and Disorders, China International Science and Technology Cooperation base of Child development and Critical Disorders, Chongqing Key Laboratory of Pediatrics, 136, Zhongshan 2nd RD, Yuzhong District, 400014 Chongqing, China

**Keywords:** Thygeson's superficial punctate keratitis, Children, Diagnosis, Management, Case report

## Abstract

**Background:**

Thygeson’s superficial punctate keratitis (TSPK) is reportedly a rare disease with an insidious onset, numerous remissions and exacerbations, and a long duration. The corneal lesions are elevated, whitish–grey in colour, and granular in the intraepithelium. A few reported cases of TSPK exist, and paediatric experience is limited. Due to the unknown aetiology and controversial treatment strategies for TSPK, we performed a literature review to summarize the criteria for the diagnosis, treatment and prognosis of TSPK to provide a basis for the treatment of TSPK in paediatric patients.

**Case presentation:**

The clinical course of a boy with TSPK who repeatedly presented with episodes of tearing, photophobia and foreign body sensation in both eyes is described. Irritation was uncontrollable with antiviral and antibiotic medications, and it was managed by corticosteroids. No recurrence was reported at the 1-year follow-up after corticosteroid replacement and tapering.

**Conclusions:**

The clinical features, treatment and prognosis between adult and paediatric TSPK patients have many similarities. The diagnosis of TSPK in children is more difficult, leading to missed diagnosis. TSPK needs to be carefully differentiated from other types of keratitis, especially intraepithelial secondary and other infectious ocular surface diseases.

## Background

Superficial punctate keratitis presents as transient, rough, corneal epithelial and primarily bilateral lesions [1]. In 1963, in recognition of Dr. Thygeson’s contributions, this condition was formally named Thygeson’s superficial punctate keratitis (TSPK). Patients with TSPK may suffer from repeated symptoms, including corneal irritation and reduced vision. The keratitis is variable with an insidious onset, numerous remissions and exacerbations, and a long duration, usually without serious sequelae. The multiple intraepithelial corneal lesions are elevated, whitish–grey in colour, and granular without stromal involvement or corneal oedema, occur predominantly in the centre of the cornea and are rarely accompanied by conjunctival inflammation. Some opacities, which can be stained with fluorescein, are the main cause of photophobia and the foreign body sensation.

Currently, there is no clear evidence that viruses or bacteria are the cause of this keratitis [2]. An immune response associated with the histocompatibility antigen HLA DR3 may be the probable cause of keratopathy [3]. The pathogenesis of TSPK is still being explored. Antibiotic and antiviral therapies are ineffective. Topical corticosteroids are widely recognized as an effective treatment for TSPK, [4] but recurrences are common once the steroids are withdrawn. In addition, the prolonged course of the disease may be related to the use of steroids [5]. Recently, immunomodulatory agents have shown potential for improving symptoms and signs, with good tolerance and few side effects. However, recurrence remains a problem. Other treatments, such as therapeutic soft contact lenses and photorefractive keratectomy, have been indicated to be beneficial for some people [6]. Optimal therapies for this disease, especially different therapies for children and adults, are still being explored. Few reported cases of TSPK exist, and paediatric experience is limited. We describe a paediatric case and performed a literature review to summarize the criteria for the diagnosis, treatment and prognosis of TSPK to provide a basis for the treatment of TSPK in paediatric patients.

## Case presentation

A 7-year-old boy (born in 2011) was referred to the Outpatient Department of Ophthalmology at our tertiary care facility in July 2018 for photophobia, tearing and blurred vision in both eyes. This was the third attack. During the events, he reported no fatigue and did not have systemic diseases. In addition, he denied contact with anyone with influenza or a cold. No indications were noted in his birth history, growth history and family history.

He had a history of ocular irritation in both eyes twice. The first time was 4 months prior (in March 2018). The boy suffered from ocular pain, tearing and photophobia for 1 month. The ocular examination showed pin-like nebula in both corneas. In addition, he was diagnosed with viral keratitis. The symptoms were not significantly relieved after 2 week of local antiviral treatment. However, his symptoms dramatically disappeared after the withdrawal of the eye drops. No follow-up was performed because the patient was symptom free. The second attack occurred 2 months ago (in May 2018). The main complaints were photophobia, tearing, and a foreign body sensation, and the boy was observed repeatedly rubbing his eyes. The ocular examination showed anterior lid margin telangiectasia and scattered punctate nebula in the centre of the cornea. The boy was diagnosed with blepharokeratoconjunctivitis (BKC) and prescribed erythromycin and tobramycin dexamethasone. The symptoms were significantly relieved, and the cornea was transparent after 1 month.

Smears and cultures of bacteria and fungi and a polymerase chain reaction (PCR) test for herpes simplex virus were performed during the first visit at the local hospital, and the results were negative.

**Fig. 1:**
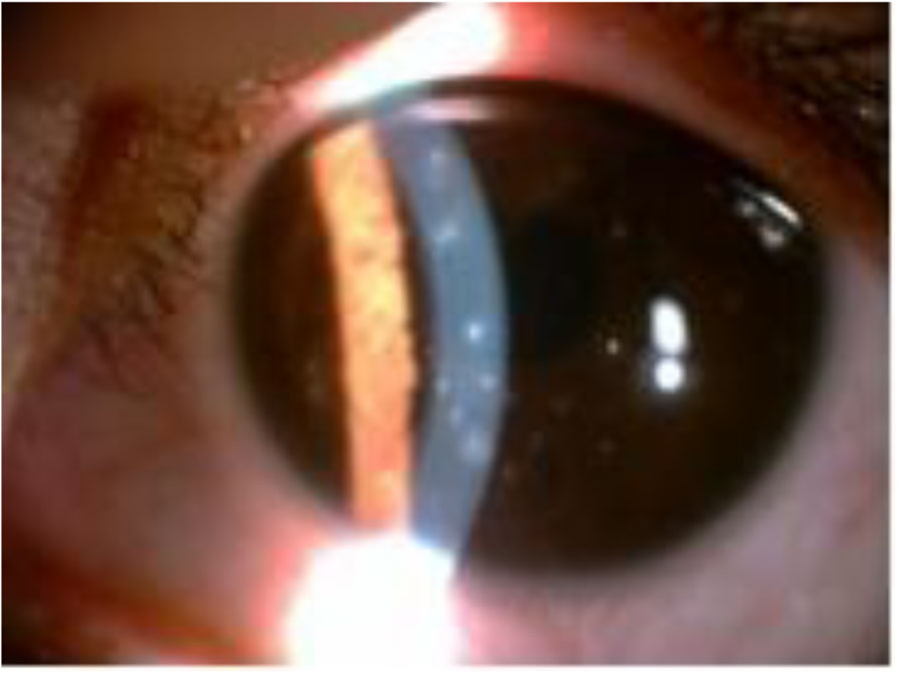
Slit-lamp photograph showing multiple discrete epithelial lesions (OS)

**Fig. 2:**
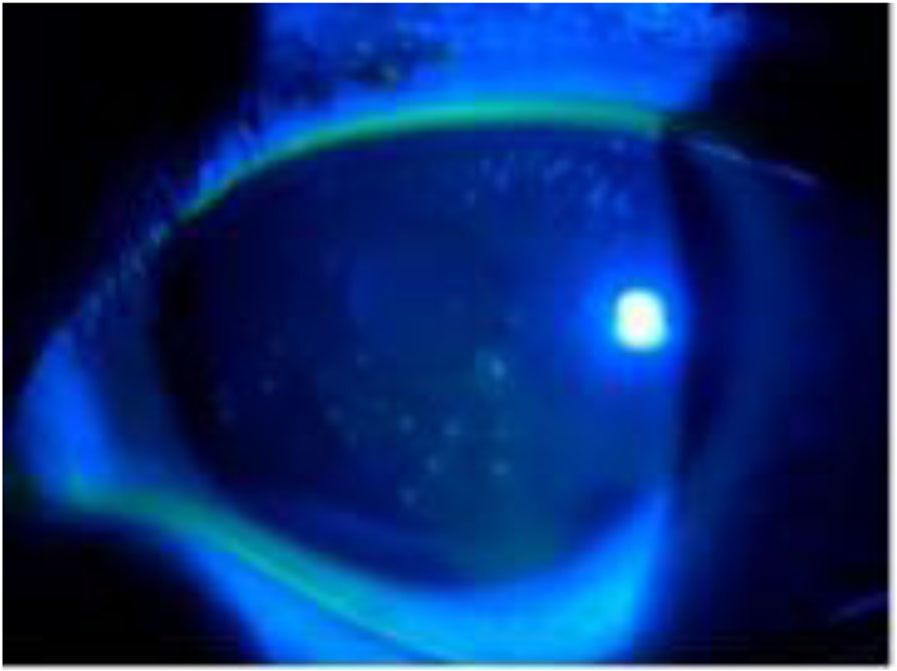
Slit-lamp photograph showing a cornea stained by fluorescein (OS)

The ocular examination performed during this visit revealed that the best-corrected visual acuity (BCVA) of the right eye (OD) and the left eye was 20/20 and 20/25, respectively. The anterior segment, including the lens, anterior chamber and palpebral conjunctivae, appeared normal in the slit lamp examination. Multiple slightly raised lesions were observed in the pupillary area within the epithelium of both corneas. These lesions were whitish-grey, coarse, and oval-shaped, and some lesions were stained with fluorescein (Figs. 1 and 2). The lesions involved the epithelium and subepithelium without invasion of the stroma or endothelium. The corneal sensitivity was intact and equal in each eye according to a cotton swab test. The tear breakup time (TBUT) in both eyes was reduced to 6 seconds. The IOPs were 17 mmHg (OS) and 18 mmHg (OD). The eye movements were normal in both eyes (OU). Abnormalities were not found in the posterior segment evaluation.

The boy was diagnosed with active TSPK in OU. Tobramycin dexamethasone drops, 4 times per day, were prescribed for 7 days. The patient’s symptoms of tearing, photophobia, and foreign body sensation were relieved at the one week follow-up visit. His BCVA was restored to 20/20 OU. Although fluorescein sodium staining remained in the cornea, the number of lesions decreased. The IOPs were 18 mmHg in both eyes. In the second week, fluorometholone 0.1% (FML 0.1%) and artificial tear drops (polyvinyl alcohol), 4 times per day, were prescribed for 7 days. The boy showed good compliance with this regimen. The symptoms disappeared in the second week. The BCVAs were 20/20 OU. The corneal lesions disappeared with no fluorescein staining. The IOPs were 19 mmHg (OD) and 17 mmHg (OS). During this visit, the child and parent were ordered to gradually reduce the frequency of FML 0.1% to 3 times a day for the first week, 2 times a day for the second week, and once a day for the following 6 months. They were requested to attend follow-up examinations in 1 month, 3 months, 6 months and 12 months. There was no recurrence after the withdrawal of FML 0.1%, and the IOPs were normal in both eyes at the one-year follow-up visit.

## Discussion and conclusions

Because of the rarity of this disease, there are no existing multicentre studies or randomized controlled trials. Retrospective case reports and case series of TSPK have been reported mostly in adults. We reviewed the current literature regarding TSPK and extracted and analysed data to provide evidence for the diagnosis and treatment of TSPK in children.

### Presentation

According to the clinical manifestations in Table 1, the most common symptoms in patients with TSPK are eye irritation (48.8%) characterized by a foreign body sensation and pain, followed by photophobia (41.9%), blurred vision (36.0%), and tearing (15.1%), and rarely accompanied by redness [7]. A lack of symptoms (2.3%) may occur during the early stages of the disease, [8] and decreased vision (19.8%) occurs when the condition worsened [9]. Only one report mentioned the symptoms of dryness (2.3%) and diplopia (1.2%) [4]. The data in Table 2 show that most cornea lesions were present bilaterally (62.7%) at the beginning of the disease [10]. In a few cases, unilateral lesions appearing soon after onset might develop bilateral involvement. Very few patients had only unilateral lesions [11]. The results differ from the signs of most viral keratitis cases, which usually begin with unilateral lesions. Patients of all ages have been reported [5]. We reviewed the literature and summarized the age-related characteristic of patients in Table 2. Of the 241 patients with TSPK, only 10 were children, 77 were adults, and the remaining 154 had no age-related information. The incidence of TSPK is lower in children (4.1%) than adults (32.0%). We are unsure of the reason causing the incidence to be lower in children. TSPK in children is likely overlooked because of poor communication. In addition, no studies have described the differences in the degrees and duration of TSPK between children and adults. Due to some age-related immunological specificities, we speculate that TSPK in Children differs from that in adults in some ways. According to the statistics in Table 2, there is no definitive gender predilection.

**Table 1:** Case reports and series about the clinical manifestation

Reported year	Author(country)	No.	Clinical manifestation								
			Irritation	Photophobia	Blurred vision	Decreased vision	Tearing	Redness	Dryness	Diploma	Asymptomatic
1980	Goldberg(America) [8]	4	4	2	1	2	1	*N/A*	*N/A*	*N/A*	*N/A*
1982	Nesburn(America) [9]	4	3	1	1	3	1	*N/A*	*N/A*	*N/A*	*N/A*
1995	Gock(Australia) [10]	1	*N/A*	1	1	1	1	*N/A*	*N/A*	*N/A*	*N/A*
2001	Fite(America) [12]	1	1	1	*N/A*	*N/A*	1	*N/A*	*N/A*	*N/A*	*N/A*
2004	Nagra(America) [4]	40	23	20	19	8	6	5	2	1	1
2004	Cheng(China) [11]	1	1	1	*N/A*	1	1	*N/A*	*N/A*	*N/A*	*N/A*
2006	Connell(Ireland) [13]	8	*N/A*								1
2007	Duszak(America) [27]	1	1	*N/A*	1	1	1	*N/A*	*N/A*	*N/A*	*N/A*
2008	Hasanreisoglu(Israel) [25]	1	1	1	*N/A*	*N/A*	1	*N/A*	*N/A*	*N/A*	*N/A*
2011	Fintelmann(America) [17]	4	2	1	8	2	*N/A*	1	*N/A*	*N/A*	*N/A*
2015	Chao(China) [28]	21	6	7	*N/A*	*N/A*	*N/A*	*N/A*	*N/A*	*N/A*	*N/A*
Total (%)		86	42(48.8)	36(41.9)	31(36.0)	17(19.8)	13(15.1)	6(7.0)	2(2.3)	1(1.2)	2(2.3)

No. number of patients, *N/A* not available

**Table 2:** Patient characteristics

	All patients	*N* = 241	N %
**Sex**	Male	119	49.3
	Female	113	46.9
	N/A	9	
**Age**	Adult	77	32.0
	Child	10	4.1
	N/A	154	
**Laterality**	Bilateral	151	62.7
	Unilateral	24	10
	N/A	66	

*N/A* not available

Almost all cases presented with the same signs [13]. The classic corneal lesions in this type of active keratitis consist of multiple and discrete punctate lesions ranging from a few to many dozens. These whitish-grey lesions are coarse, oval-shaped and slightly raised favouring the pupillary area. The lesions can be stained with fluorescein. There is no abnormality in the corneal stroma, and the conjunctiva is usually free of congestion. The lesions are intraepithelial, with minimal or no subepithelial oedema and no subepithelial infiltration by slit lamp. Corneal sensation is normal.

According to in vivo laser confocal microscopy, the lesions are located in the anterior elastic layer and the anterior stroma [14]. Loss of intercellular adhesion, cell size enlargement and hyperreflectivity, considered signs of epithelial oedema and desquamation, can be observed [11, 14].

Patients may experience remissions and exacerbations. and the disease may recur for several years [17]. The longest reported course with steroid use was 41 years [17]. Most patients have no sequelae. In addition, corneal thickening, stromal opacification, and corneal scarring are the consequences of a prolonged and untreated disease course [16, 17].

### Diagnosis and differential diagnoses

The clinical symptoms and signs are the diagnostic basis for TSPK. According to Thygeson’s summary, the diagnostic features of TSPK are as follows: (1) the presence of bilateral punctate epithelial keratitis; (2) a chronic course with exacerbations and remissions; (3) healing without scar formation; (4) no response to antibiotics; and (5) a striking symptomatic response to topical corticosteroids [18]. No definitive diagnostic criteria have been proposed to date.

TSPK needs to be carefully differentiated from other types of keratitis, especially bacterial and viral keratitis. In TSPK, there is no obvious conjunctival hyperaemia or increased secretion, and bacterial culture is negative, distinguishing TSPK from bacterial infection. However, TSPK is often misdiagnosed as viral keratitis because the lesions associated with TSPK are similar to the corneal infiltrates of viral keratitis, especially during the early stage. In patients with viral keratitis, there is generally a history of weakened immunity, presenting as chills, fatigue, etc. Without intervention, the lesions in the corneal epithelium may aggravate to subepithelial oedema and subepithelial infiltration. In addition, unilateral viral keratitis is more common than unilateral TSPK.

Microsporidial keratoconjunctivitis presents with corneal lesions similar to that of TSPK. However, it is accompanied by mild non-suppurative papillary or follicular conjunctivitis, and it is mostly unilateral. Moreover, a positive smear for microsporidial spores is the criterion for diagnosing microsporidial keratitis [19].

Notably, children with TSPK have an obvious foreign body sensation and rub their eyes frequently, which may lead to other secondary infectious ocular surface diseases. In the current case, during the second visit, the child was misdiagnosed with BKC because of the presence of blepharitis. Blepharokeratoconjunctivitis (BKC), which is usually caused by gram-positive organisms, is an infectious disease that is manifested by inflammation of the eyelids and the front of the eye [20]. Coagulase-negative staphylococci, Pseudomonas aeruginosa and Staphylococcus aureus are the common pathogenic organisms. The symptoms of children with BKC include tearing, itching and red eyes. Cornea scars can develop if not treated effectively for a long time, causing the loss of vision.

### Aetiology

Although the cause of TSPK is still unclear, there is an association with viral infection and the immune mechanism associated with HLA DR3.

In early TSPK investigations, most researchers agreed that the cause of TSPK was viral. In 1950, Braley and Alexandra first isolated a virus from a case of superficial punctate keratitis in a rabbit [21]. However, the same conclusion has not been subsequently reached. In 2003, Reinhard harvested epithelia cells from 9 patients with TSPK and failed to amplify DNA of the varicella zoster virus (VZV) genome [22]. In 2007, Connell reconfirmed that viruses (HSV 1, HSV 2, VSV and adenovirus) were not responsible for TSPK [13]. A case of superficial keratitis associated with Epstein-Barr virus (EBV) was also reported to resemble the corneal lesions of TSPK [23]. EBV keratitis mostly occurs with mononucleosis. The pathogenesis of TSPK has been hypothesized to be due to an immune response induced by virus infection [9]. Furthermore, the corneal lesions of TSPK might be antigen-antibody complexes with an associated mononuclear response, and they are strikingly similar to the signs of EBV infection. Other viruses might be latent in epithelial cells in low copy numbers that cannot be detected and provoke the immune system to produce antigen-antibody complexes with an associated mononuclear response [9].

In 1981, Darrell reported that the frequency of the histocompatibility antigen HLA DR3 was significantly increased in patients with TSPK [3]. HLA-DR3 has a genetic association with TSPK. It has been confirmed that T cell-related immune mechanisms play a role in TSPK. Furthermore, immunosuppressive agents, including cyclosporin A and tacrolimus, have been reported to be effective in relieving the symptoms of TSPK, [18] supporting the theory of an immunological component to this condition. Age-related immunological peculiarities may cause different response to treatment with immunomodulation agents [24]. Different immunologic mechanisms my occur in children versus adults. To date, there are only a few relevant studies, [3] and more studies are needed to provide evidence.

### Management

#### Antibiotic and antiviral therapy

Approaches for the management of TSPK are still being explored. We summarized the TSPK case reports and series regarding treatments and prognoses in Table 3. Older studies have shown unsatisfactory outcomes with antibiotic and antiviral therapies [2]. In 1981, Tabbara studied 45 cases of TSPK and found that antiviral therapy and topical antibiotics had no effect on the course of the disease [5]. In 1982, Nesburn treated four patients with TSPK with 1% trifluridine drops and found that trifluridine alleviated attacks of TSPK [9]. Unfortunately, only four patients received combined therapies including topical corticosteroids.

**Table 3:** Case reports and series of TSPK about treatment and prognosis

Reported year	Author(country)	Total	No.	Treatment	Prognosis
1966	Thygeson(America) [3]	27	7	Steroid	Effective in suppressing symptoms and the epithelial opacities.
			4	IDU(5-iodo-2’-de oxyuridine)	Ineffective
1979	Abbott(America) [18]	1		Steroids and Artificial tears	Remission and recurrence for 16 years
1979	Forstot(America) [25]	3	1	Steroids	Relief of symptoms and good visual acuity for over 2 years
			1		Dependent on corticosteroids.
			1	Soft contact lenses	Symptomatic relief
1980	Goldberg(America) [9]	4		Soft contact lenses	Resolution of the lesions and relief of discomfort.
1981	Tabbara(America) [6]	45	32	Steroid	50% of the cases, exacerbation were controlled
			2	Soft contact lenses	Relieve the symptoms and improve the visual acuity
			10	Scrapings	No effect on the course of TSPK.
			20	IDU and Antibiotics	No effect on the course of TSPK.
1984	Nesburn(America) [10]	4		Trifluridine therapy	Symptoms and later the signs of the disease disappeared
1999	Reinhard(Germany) [23]	17		Cyclosporin A 2%	71.5% of cases,complete suppression
2002	Reinhard(Germany) [22]	28		Cyclosporin A 2%	90.5%case improvement
2001	Fite(America) [12]	1		PRK	Corneal lesions recurrence outside the laser ablation zone
2002	Goldstein(America) [8]	1		PRK	No recurrences
2004	Nagra(America) [5]	40	35	Steroids	Symptomatic relief
			2	Artificial tears	N/A
			1	Soft contact lenses.	N/A
2004	Netto(Brazil) [26]	1	OD	PRK	No recurrences
			OS	LASIK	Recurrence after 10 month
2007	Duszak(America) [27]	1		Steroid	Signs and symptoms disappeared
2008	Hasanreisoglu(Israel) [15]	1		Cyclosporin A 0.5%	Symptom resolution and corneal clearing
2011	Fintelmann(America) [16]	4		Steroids	Symptomatic relief and reduce the risk of corneal scarring.
2015	Chao Jiang(China) [28]	21		Steroids combined with antiviral drugs	17/21cured and 4/21 recurrence
2015	Marquezan(Brazil) [29]	14		Tacrolimus 0.03% eye ointment	Improved VA, symptoms, and signs

*IDU* 5-iodo-2’-de oxyuridine, *N/A* not available

#### Topical steroids

In 1870, Thygeson reported the relief of symptoms with a hydroxymesterone (HSM) 1% suspension [2]. In 2003, Nargra followed 40 patients with TSPK for 10 years and suggested that a similar low-dose steroid had a positive effect on the management of TSPK [4]. In 2012, Fintelmann found that the use of topical corticosteroids alleviated the corneal inflammation and reduced the risk of corneal scarring, especially in patients with a longer disease duration [17]. Notably, steroids require longer-term, low-dose, regular use in patients with this disease. It is also essential to provide detailed education to patients.

Topical corticosteroids represent the mainstay of the treatment for TSPK. However, it is believed that steroids might contribute to prolonging the course of the disease, and at least one recurrence is common. The side effects of topical corticosteroids include secondary glaucoma, cataracts, and susceptibility to infection, and topical corticosteroids may delay the repair of the corneal epithelium.

#### Immunomodulatory agents

In recent years, cyclosporine A and tacrolimus have been increasingly used in the clinic due to their immunomodulatory effect. Compared to topical steroids, they are safe without side effects.

Holsclaw first applied cyclosporine A, which reduces interleukin-2 production by T-lymphocytes, leading to the inhibition of T cell proliferation, for the treatment of TSPK [15]. In 1997, Reinhard carried out a prospective study to demonstrate that cyclosporine A 2% inhibited the typical epithelial and subepithelial opacity associated with TSPK in most cases [24]. Children seemed to be less responsive to topical cyclosporine A than adults. Age-related immunological specificities supposedly play a large role in this difference. In 2008, Hasanreisoglu reported that topical cyclosporine A 0.5% was applied to a 15-year-old boy for 4.5 years [25]. In 2015, Marquezan retrospectively diagnosed 14 patients with TSPK. Tacrolimus successfully improved visual acuity, symptoms and signs in all patients with no side effects.

Unfortunately, attempts to withdraw these immunomodulatory agents resulted in recurrent disease. Long-term low-dose treatment is necessary in such cases.

#### Other treatments

Other treatments include therapeutic soft contact lenses and photorefractive keratectomy. In 1980, Goldberg found that the symptoms were resolved after bandaging, and the patients who used therapeutic soft contact lenses reported relief from discomfort [6, 8]. Even though soft contact lenses relieved pain, they impaired visual acuity via an irregular anterior corneal astigmatism, and soft contact lenses cannot cure the disease. Lesions were still present once the lenses were discontinued. Moreover, these lenses are not available to everyone. Microbial infection and mechanical damage may be potential complications.

Photorefractive keratectomy has been considered an alternative therapeutic approach for patients with myopia and TSPK [12]. The mechanism is unknown. Artificial tears are often used as an adjunctive medication during treatment; [16] however, this treatment only relieves the foreign body sensation caused by a dry cornea.

## Conclusions

The diagnosis of TSPK is based on the clinical symptoms and signs. Ocular irritation, including a foreign body sensation and pain, is the most common symptom in patients with TSPK, followed by photophobia, blurred vision, and tearing, and TSPK is rarely accompanied by redness, dryness and discharge. Multiple and discrete punctate corneal lesions are coarse, oval-shaped and slightly raised in the intraepithelial tissue, without subepithelial oedema and no subepithelial infiltration. In paediatric cases, the diagnosis of TSPK needs to be differentiated from bacterial and viral infections on the ocular surface. In particular, secondary infections of TSPK are easily misdiagnosed. The aetiology is still unclear, but vast evidence refutes microbial infection. Immune mechanisms may play a role in TSPK; however, further basic research is needed to elucidate these mechanisms. Topical steroids represent an effective therapy. Immunomodulatory agents have a good advantage in avoiding side effects according to the previous literature, but their safety in children remains unclear. Soft contact lenses, as a supplement, can rapidly resolve the lesions and relieve discomfort. Because TSPK is a recurrent disease, it is very important to provide detailed education to patients. There are many similarities between adult and paediatric TSPK. In our case, the clinical feature, treatment and prognosis were consistent with the characteristics of TSPK in adults. According to the data from the current paper, the incidence of TSPK in children is much lower than that in adults. Children’s poor communication and noncooperation during the ocular examination render the diagnosis of TSPK more difficult, resulting in missed diagnosis. Furthermore, there may be different immunologic mechanisms between children and adults because of age-related immunological peculiarities. More paediatric cases are needed to assess the mechanism of paediatric TSPK.

## Data Availability

All data generated or analysed during this study are included in this published article.

## Abbreviations

TSPK: Thygeson's superficial punctate keratitis
BKC: Blepharokeratoconjunctivitis
IOP: Intraocular pressure
BCVA: Best-corrected visual acuity
OU: Both eyes
OD: Right eye
OS: Left eye
